# Performance Evaluation of an Enhanced Uplink 3.5G System for Mobile Healthcare Applications

**DOI:** 10.1155/2008/417870

**Published:** 2008-12-31

**Authors:** Dimitris Komnakos, Demosthenes Vouyioukas, Ilias Maglogiannis, Philip Constantinou

**Affiliations:** ^1^Mobile Radio Communications Laboratory, School of Electrical and Computer Engineering, National Technical University of Athens, Zografou, 15773 Athens, Greece; ^2^Department of Information and Communication Systems Engineering, University of the Aegean, Karlovassi, 83200 Samos, Greece; ^3^Department of Biomedical Informatics, University of Central Greece, Papasiopoulou 2–4, 35100 Lamia, Greece

## Abstract

The present paper studies the prospective and the performance of a forthcoming high-speed third generation (3.5G) networking technology, called enhanced uplink, for delivering mobile health (m-health) applications. The performance of 3.5G networks is a critical factor for successful development of m-health services perceived by end users. In this paper, we propose a methodology for performance assessment based on the joint uplink transmission of voice, real-time video, biological data (such as electrocardiogram, vital signals, and heart sounds), and healthcare records file transfer. Various scenarios were concerned in terms of real-time, nonreal-time, and emergency applications in random locations, where no other system but 3.5G is available. The accomplishment of quality of service (QoS) was explored through a step-by-step improvement of enhanced uplink system's parameters, attributing the network system for the best performance in the context of the desired m-health services.

## 1. INTRODUCTION

Promising high-speed mobile networks enable the deployment of new advanced services, facilitating the development of emerging services in the electronic healthcare area. Obstacles for healthcare services are time and space between the providers and the patients. Wireless technology came to encompass the e-health monitoring everywhere from any given location, providing the so-called m-health services. The benefits of wireless technology have been illustrated in a number of different examples and cases [1], especially for mobile applications [2].

During the last years, there has been increased research efforts on the production of commercial mobile health systems based on Wireless Fidelity (WiFi), General Packet Radio Service (GPRS), and 3rd Generation Universal Mobile Telecommunications System (3G UMTS) networking technologies [3]. The introduction of high-speed data rate, wide bandwidth, digital and encrypted communication technology makes possible the delivery of audio, video, and waveform data to wherever and whenever needed. It is hoped that the current deployment of 3G-based systems with global operational morphologies will improve some of the limitations of the existing wireless technologies and will provide a well-organized platform for mobile healthcare services.

In emergency cases, where immediate medical treatment is the key issue, studies revealed that early and specialized prehospital patient management contributes to the patient's major possibility of continued existence [4, 5]. Especially for the cases of serious injuries [6], the way the incidents are treated and transported on the way to the hospital is crucial for the survival of the patients. For example, in case of car accidents, emergency transportation and real-time diagnosis are the most vital parameters for giving the precise rehabilitation during the critical minutes toward the hospital and increasing the possibilities of surviving. Thus, people in ambulances who are the first to handle these emergency situations do not always have the required advanced background and experience to manage sufficiently all cases. Wireless transmission of vital biological signals and scene video of the patient along with video conference with an experienced doctor at the hospital can achieve proper first aid of the patient. Nevertheless, collaborative teleconsultation by moving physicians, in terms of retrieving significant healthcare records, is imperative and in most of the cases valuable.

In this context, high speed packet access (HSPA) for uplink and downlink, together with 3G and 4G systems, such as WiMAX [7, 8], is expected to enforce the m-health applications and overcome the boundaries between time and space. Previous studies of 2G [9] and 3G networks [10, 11] showed performance assessment that aims to support m-health services evaluating the end-user perceived service performance, in relation to the performance of a 2G and 3G network, in order to improve the end-to-end delay characteristics of the telemonitoring service, as well as to optimize the throughput behavior. High usability, support for multimedia services with good reliability and low-transmission cost, personalization of the services, more capacity, and spectrum efficiency are some of the key features of the evolving mobile technologies. Such technologies will make available both mobile patients and end users to interactively get the medical attention and advice they need, when and where is required in spite of any geographical obstructions or mobility constrains.

The scope of this study was to design and evaluate via simulation an integrated high-speed uplink mobile telemedicine system for emergency and near-emergency conditions. HSPA is an evolving mobile technology newly deployed and commercially utilized by a small number of operators so far, thus the need for system simulation was demanding prior to real-time operation. The simulation trials were selected to represent a range of services and to include both asynchronous and synchronous applications (nonreal-time, near real-time, and real-time requirements) in medical data transmission. The overall goal was to test the ability of 3G and beyond 3G infrastructures to support value-added services. The trials were evaluated in terms of admission control and quality of service (QoS) provisioning, the total throughput that the network can accomplish versus the category of patient condition, the priority of the patient in case of an emergency, the data rate, and the congestion of the 3.5G network, planned for uplink circumstances. Moreover, various simulation scenarios were implemented in order to incorporate the typical m-health scenarios for high-speed data transfer.

The remainder of the paper is organized as follows; in Section 2, the evolving technology of the HSPA is described in relation to the general m-health requirements, while in Section 3, the specific operational m-health scenarios under study are presented. Section 4 includes the description of the developed simulation model, whereas in Section 5, experimental results regarding patient condition (emergency or simple patient monitoring), making use of m-health services session dedicated to video application, VoIP, web browsing, medical and file data transfer, are presented. The main objective of these experiments is the validation of the system throughput for the aforementioned scenarios, the delay of the packets transmission, the jitter and the latency of the system, and the overall congestion of the cell. Finally, Section 6 discusses the findings and concludes the paper.

## 2. NEXT GENERATION WIRELESS TELEMEDICINE TECHNOLOGY AND REMOTE HEALTHCARE REQUIREMENTS

The evolving technology for 3G is called HSPA which is a packet-based cellular system deployed on top of the current WCDMA networks. HSPA is an evolution of WCDMA-UMTS technology, achieving greater bit rates and reduced delays. Responsible for the standardization of HSPA is the 3GPP organization [12–14]. The commercial utilization of this technology is rather new, since new HSDPA (downlink) networks are launched by European providers continuously, while the first enhanced uplink (HSUPA - uplink) networks were implemented only during the last year. Theoretically, on the downlink the maximum achieved bit rate is about 10.7 Mbps using 16-QAM modulation, while on the uplink the maximum bit rate exceeds 5.5 Mbps per base station (Node-B). Currently, 3.6 Mbps and around 1.5 Mbps are the common peak bit rates provided by mobile providers for downlink and uplink, correspondingly. HSPA incorporates a significant number of innovative features, such as adaptive modulation and coding, short transmission time interval (2 milliseconds), fast hybrid automatic repeat request (HARQ), customized schedulers for the proper manipulation and routing of the data, as well as the possibility for a multiple input multiple output (MIMO) add-on. The uplink characteristics of HSPA are depicted in Table 1.

**Figure 1:**
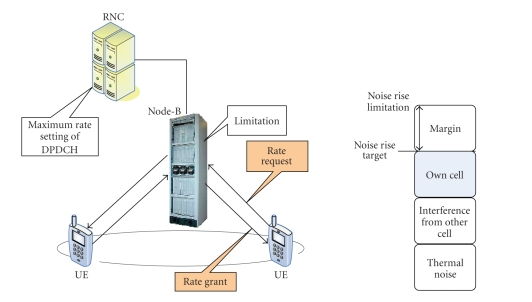
Node-B scheduling and target noise rise.

Figure 1 depicts the enhanced uplink architecture and the interconnection of the fundamental elements, such as the radio network controller (RNC), the 3G base station (Node-B), and the user equipments (UEs). The main characteristics of fast Node-B scheduling are depicted during the uplink session and the required procedures between the UE and the Node-B, which is responsible for controlling the transmission rate and assigning of UE. The scheduling process is performed by Node B in order to make the noise rise (signal-to-noise power) of a required level. Layer 1 (L1) signal of Node-B limits the maximum rate and therefore the power offset for dedicated physical control channel (DPCCH). Since the control delay is shorter than that of RNC, adaptive control is performed in association with noise rise fluctuation. UE sends control signals for uplink as rate request to Node-B, while Node-B returns UE L1 signals as rate grant. L1 signal from UE for rate request has a rate-increasing requirement based on the total buffer size. UE ends Layer 2 signal as rate request including buffer size of the highest priority data flow and the total buffer size. Downlink control signals are absolute grant (AG) and relative grant (RG). AG means the absolute value of the power offset permitted for the power usage, thus Node-B, that controls a serving cell, can send AG. No AG is transmitted from Node-B that controls nonserving cells. RG is used for controlling fluctuations for power offset and is sent from all cells in enhanced uplink neighboring cells.

The ability of HSPA to serve simultaneously a sufficient number of mobile terminals running multiple demanding applications while keeping the end to end delay low makes it an ideal candidate for a vast number of new services. Due to the increased capacity that HSPA is able to offer and its packet-based nature, the implementation of a variety of applications over cellular networks is now feasible [15, 16]. Considering the constant geographical expansion and technical development of HSPA networks, they should be soon ready to credibly host e-health services, emphasizing on mobile e-health, emergency and patient followup applications.

Electronic healthcare applications, including those based on wireless technologies span the areas of emergency health care, telemedicine in various forms (telecardiology, teleradiology, telepathology, teledermatology, teleophthalmology, and telepsychiatry), and electronic access to health records. The range and complexity of telecommunications technology requirements vary with specific medical or health applications. Except for medical images and running through full motion video, the majority of biosignal medical devices require relatively low-data transmission rates [17, 18].

Regarding the transmission of medical images, there are essentially no theoretical bandwidth requirements, but lack of bandwidth needs longer transmission time. Yet, high-quality medical images such as a single chest radiograph may require from 40 to 50 Megabytes. In practice, it is desirable to transmit medical images during a single patient visit, so as to at least avoid a followup visit. Medical image compression techniques have primarily focused on lossless methods, where the image has to be reconstructed exactly from its compressed format due to the diagnostic use.

In regards to the digital video compression, the digital imaging and communications in medicine (DICOM) committee has not yet adopted any standard. The adoption of MPEG-2 is possible, but this is limited by the MPEG-2 requirement for constant delay method for frame synchronization. On the other hand, the transmission of offline video is still possible. It is important to distinguish among the requirements for real-time video transmission, offline video transmission, medical video and audio for diagnostic applications, and nondiagnostic video and audio. Real-time video transmission for diagnostic applications is clearly the most demanding. Offline video transmission is essentially limited by the requirement to provide patient doctor interaction. Real-time diagnostic audio applications include the transmission of stethoscope audio, or the transmission of the audio stream that accompanies the diagnostic video. A typical application will require a diagnostic audio and video bit stream, in addition to a standard teleconferencing bit stream [19, 20].

Future challenges in m-health systems are already mentioned in [21], and were partially covered in this study. In general, m-health applications may be categorized in two groups depending on the required transmission mode:

- real-time applications: these are referred to multimedia connections between centers and moving vehicles including audio and video exchange, biomedical signals, and vital parameters transmission, such as electrocardiogram (ECG) signal, blood pressure, oxygen saturation, and so forth;
- near real-time applications: these correspond to applications enabling access to administrative files and electronic patient report (EPR) transfer (from medical data exchange between centers and moving vehicles or specialty sections), clinical routine consults during accesses to databases, queries to medical report warehouse, and so forth.

In this study, we deal with applications from both groups, used in emergency situations and for teleconsultation purposes, respectively. Therefore, the QoS requirements for the discussed m-health services are set to the highest level for emergency and medium to low level for near real-time applications, as depicted in Table 2. The corresponding m-health scenarios are discussed in Section 3.

## 3. 3.5G m-HEALTH SCENARIOS

Wireless mobile systems may realize various m-health services. Most of them are already covered, but due to the extended performance of the enhanced uplink in terms of throughput, new m-health services were implemented via simulation procedure. Thus, in this section we initially discuss potential scenarios of mobile healthcare services, incorporating emergency real-time and near real-time applications.

### 3.1. Emergency services in case of accidents

This service refers to the support of transport healthcare units (i.e., ambulances) or primary care units (i.e., rural centers) in case of accidents. Recent studies conclude that early and specialized prehospital patient management contributes to emergency case survival. Especially in cases of serious injuries of the head, the spinal cord, and internal organs the way of transporting and generally the way of providing care is crucial for the future of the patient. Unfortunately, general practitioners in remote health centres or ambulance personnel, who usually are the first to handle such situations, do not have the required advanced theoretical knowledge and experience. Since, for practical and financial reasons, primary care or transport healthcare units cannot be staffed by specialized physicians, general doctors can only rely on directions provided to them by experts. An m-health service in this case allows specialized physicians located at a hospital site to coordinate remote-located primary care or ambulance services paramedical staff via telediagnosis and interactive teleconsultation means. Table 3 summarizes the typical data transmitted in emergency services in case of accidents.

### 3.2. Teleconsultation collaborative sessions between moving physicians

Recent developments in networking and computing technologies and the expansion of the electronic health record system have enabled the possibility of online collaboration between geographically distributed medical personnel. In this context, a teleconsultation session implements a collaborative working environment for physicians in dispersed locations, by enabling (a) electronic exchange of medical data, (b) voice/video/chat communication, and (c) common workspace management (i.e., common image processing toolbox, annotations, etc.) (see Table 4) [22]. In case one at least of the commuting doctors is moving or in a random location with no availability of fixed networks, a 3.5/4G platform may be used as a communication medium.

### 3.3. Medical information management service—mobile access to electronic health records (EHRs)

This service is related to applications, enabling the mobile ubiquitous delivery of medical data and implementations of mobile electronic health records (EHRs), accessible by PDAs or tablet PCs. This service is provided to physicians that require immediate access to patient's medical data from random locations [23]. Therefore, only broadband cellular systems (i.e., 3/4G) may be used due to the corresponding data sizes. The medical data transmitted in a mobile EHR system are depicted in Table 5.

## 4. DESCRIPTION OF THE SIMULATION MODEL

In order to evaluate the performance of the mobile healthcare applications over the 3.5G system under study (specifically high speed enhanced uplink aka HSUPA) a simulation campaign is among the optimum solutions [24, 25]. In this case, a simulation was developed using OPNET [26]. Though we will only refer to the reverse link hereafter, both forward and reverse link of the network were implemented in the simulator used. During the development of any platform, necessary simplifications have to be taken into account, usually leading to a slight overestimation of the system's performance. All the fundamental elements (RNC, Node-B, air interface, UE, etc.) of a complete cellular network were simulated, to such an extent for each one, so as to be precise enough for our scope. There is also a traffic generator process, which simulates several applications as will be mentioned later on.

The model consists of a single hexagonal cell, while the first layer of six identical adjacent cells has been virtually deployed (i.e., handover is not allowed and mobile terminals are restricted within the coverage area of central cell) in order to accurately incorporate interference effects. All the attributes of the network were adjustable; therefore a variety of scenarios may be investigated. The sessions may be statically created (e.g., at the beginning of a simulation run) or dynamically inserted into the network using equivalent distributions (exponential, Poisson) and may run one or multiple services. Mobility has been taken into account for each terminal, corresponding to vehicular and pedestrian profile [27]. The propagation model calculates path loss, shadowing, and small scale fast fading, adapted to a dense urban environment. Shadowing is both spatially and angularly correlated. Rayleigh fading is considered [28], whereas Doppler power spectral density is modelled by the well-known Jakes formula [29]. Channel samples are generated offline through a Matlab implementation of the above channel model. A detailed methodology for extracting these samples is described in [30]. As enhanced uplink is an interference limited system, calculations for the admission of a new session into the network and power allocation per time transmission interval (TTI) also exist.

The traffic process is capable of generating all the typical applications running over a cellular network. VoIP, web browsing, video application, and FTP are typical examples of applications provided. Some of these were simulated based on their standards (e.g., VoIP), while for others trace files from real networks were used (e.g., H.263 codec medical video) [31]. Different load mixes may be examined, as the percentage of each service in the total load is arranged for each simulation run. In this performance, evaluation simulation campaign, medium to highly congested cell is studied. VoIP is considered to incorporate all possible enhancements, resulting to a bit rate of 12.2 kbps per user, with an adaptive multirate (AMR) codec. Video applications codec is either based on H.263 or MPEG-4 standards. Web browsing is a common bursty source, while FTP service is a source of constant bit rate. Congestion control will shortly detect a build-up delay (or lost packets) and inform the Node-B about it. The scheduler is responsible to maintain uplink interference below the threshold level, by signaling the mobile terminals to adjust their transmission power. It is evident from the results of the simulation procedure that the end-to-end delay per application for the VoIP and video sessions is quite low and that enhanced uplink is able to withstand the meet of service's requirements [32].

Also, several necessary simplifications were taken into account. First of all, after the establishment of a session, the only part which is able to terminate the connection is the UE. This means that the network is not able to drop a session for any reason (e.g., congestion of the network). A single cell network was used throughout the paper and all UEs were bounded to move within the area of coverage of this cell (if a UE leaves this area, the mobility process will force it to reenter). Also, no erroneous packet reception was considered and no packet discard was allowed, leading to a slight loss in the quantitative (but no qualitative) accuracy of the results. Considering the aforementioned setup, the simulator's environment and results are as realistic and accurate as possible. The key simulator's parameters are summarized in Table 6.

## 5. SIMULATION RESULTS AND NETWORK EVALUATION

As discussed in Section 3, three primary scenarios were taken into consideration, and a step-by-step performance evaluation was accomplished arranging the network for serving a number of possible occasions. We kick off by examining the requirements for serving emergency cases, continue by studying collaborative sessions between moving physicians and finally the scenario of ubiquitous mobile access to electronic health records is presented. Results are mainly presented in terms of end-to-end delay, as this metric is widely considered to be amongst the optimum indicators in order to evaluate a network's performance, influencing directly or indirectly most of the other performance metrics. During these scenarios, significant contributions for the optimization of a remote healthcare-oriented enhanced uplink network were exhibited.

### 5.1. Simulation results and proposition for emergency services in case of accidents

In order to extract conclusions about the overall performance of enhanced uplink, the first test case to be examined for this scenario is the concurrent existence of two emergency sessions in the network. Table 7 depicts the parameters of this simulation run, while the selected services and bit rates correspond to those of Table 3.

**Figure 2:**
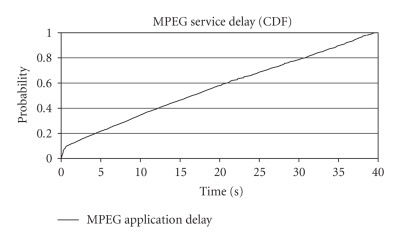
MPEG application delay for first test case of the first scenario (CDF).

The two established sessions require approximately 2 Mbps in total, while during video bursts this value may rise even 20% or more. An important factor was that no packet drop was allowed, no matter the delay until the packet was finally routed. Figure 2 is the most characteristic graphic result of this case study, showing the probability of the delay in terms of CDF (cumulative distribution function) versus time.

It is evident from Figure 2 that the network is not able to cope with two simultaneous emergency sessions. The delay of MPEG video service is constantly increasing, while this trend shows no tendency to decrease or at least stabilize. At this point, we should conduct a brief analysis of the network's structure and capabilities. Due to enhanced uplink's nature, the allocation of excessive bit rate (above 1 Mbps) to a single session raises a series of issues. enhanced uplink is—as far as its capacity is concerned—an interference limited system. This interference caused by every emission is additive and the main sources contributing to the build up of this summation were the UE's of the cell, which utilized the system's resources and the interference from other cells. By allocating high bit rate to one UE (which needs a high-power allocation to achieve this bit rate), usually this session increases dramatically the interference in the cell, consuming a large portion of the RoT margin. By exceeding or abusing RoT, another high-bit rate allocation within the same TTI is either no more available in many cases, or marginally feasible in others. This is the case here, as the first session actually blocks the second one and vice versa per TTI, leading delay to an unacceptable level.

**Figure 3:**
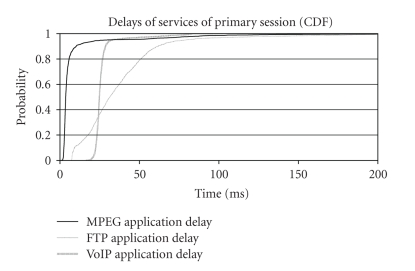
Application delay for second test case of the first scenario (CDF).

The previous conclusion will be verified along with the second test case of this scenario. The new parameters are given in Table 8. The main difference is that the second emergency session has been replaced by two new (not emergency) sessions with lower bit rate requirements each, though the aggregate bit rate requirement is approximately the same as above. The delay results are shown in Figure 3.

In Figure 3, the delay for all applications was kept in 95% of the samples below 80 milliseconds and in 98% of the samples below 150 milliseconds (hereafter the term “delay” we mean the time one packet waits in the sender's buffer to be routed). It seems that there has been a drastic overall improvement of system's performance due to the fact that each of the two new sessions does not require high-bit rate allocation. The network's throughput increased, while it also became stable. This result confirms the initial assumption that enhanced uplink cannot always sufficiently manage two demanding sessions, as total throughput is not the ultimate decisive factor. A rule of thumb could be assumed, demonstrating that remote healthcare-oriented enhanced uplink should bound bit rate allocation to 768 kbps per session, which is satisfactory for almost all typical cases. From another point of view, most contemporary commercial enhanced uplink platforms practically do not support greater bit rates (considering the fact that serving only one UE at any given time is rather seldom).

There are several solutions that may be applied to solve the aforementioned issue. Among them, the most efficient and realistic ones are

- as smooth video reception is of vital importance in emergency medical situations, the transmission bit rate should be constrained to 512 kbps, which yields to a quite adequate quality for this service.
- If a session demands more than 768 kbps and consists of two or more applications, then these applications could be automatically or manually torn down to two sessions, even decreasing in some cases the total interference caused to the network. Supposing that the equivalent building blocks exist on the network's and on the terminal's side and that appropriate user equipment is available (e.g., laptop with two access cards), such an action is possible to be implemented.
- As a last measure, if a finest quality video is considered to be necessary, then the network should be tuned in such a way, while during video bursts (or when the video delay increases beyond a threshold value), all services would be prioritized according to their significance, in order to temporarily mute low-priority services. This muting may also include VoIP if we use real-time video which includes audio stream. This is considered to be a worst case and clearly suboptimum congestion control measure.

### 5.2. Simulation results and proposition for teleconsultation collaborative sessions between moving physicians

**Figure 4:**
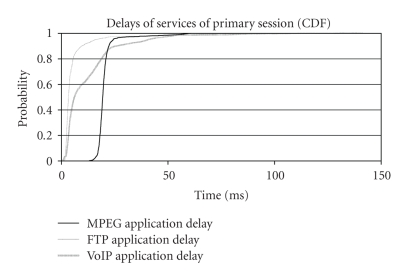
Application delay for second scenario (CDF).

**Figure 5:**
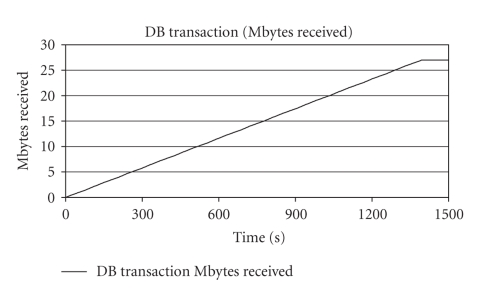
DB transaction (received MBytes) versus time for second scenario.

In this section, we studied teleconsultation collaborative sessions between moving physicians. The parameters for this simulation run are depicted in Table 9, supposing that for this kind of session, VoIP and video services of 12.2 and 512 kbps were needed. A stream service of 10 kbps for common workspace management controls was also included. Finally, during the session 27 MB of data were transmitted from a database. These data were distributed as follows: 100 kB for demographic data, 1 MB for laboratory, clinical data, and medical history, 24 MB of prerecorded medical video for diagnosis and medical images, as described in Table 4. In addition, results from this scenario are given in Figures 4 and 5.

According to Figure 4, enhanced uplink network can easily handle the traffic required to sufficiently serve all services for this scenario. The delay for each separate application running within the primary session was low, as 95% of the samples have a delay below 40 milliseconds, while 98% of the samples have a delay below 50 milliseconds. Served throughput equals actually the nominal bit rate of each application and it was extremely steady. Conclusively, enhanced uplink definitely surpasses the demands of this scenario by providing adequate quality of service for all the services of our session.

On the other hand, one problem becomes noticeable by observing Figure 5. In order to acquire the 27 MB from database, approximately 24 minutes were needed. This comes as no surprise, as from the previous scenario the maximum bit rate allocation has been bounded to 768 kbps. Due to the fact that MPEG video utilizes 512 out of the total 768 kbps, control tools 10 and VoIP 12.2 kbps, about 230 kbps remain for other data. At this speed, the time needed to acquire 27 MB is at best 16 minutes. Although the size of 27 MB is considered high, it might represent an important portion of an EHR, necessary for a teleconsultation session. Undoubtedly, a physician does not have the luxury to spend so much time for file transfer. A remedial action should be undertaken at this point. The obvious one is to place DB transaction services at a different session. This way the transaction will finish much earlier (as 27 MB divided by 768 kbps yields a result of less than 5 minutes). The interference induced by this new session to the network was not an important discouraging factor, as DB transaction is a background service. In case the platform needs the resources, they will be immediately released simply by temporarily cutting off the DB transaction service (the other session containing VoIP + controls + MPEG video will not be affected by the interrupt). After the transaction's end, the resources will be returned to the network in order to be reused.

### 5.3. Simulation results and proposition for medical information management service—mobile access to electronic health records

**Figure 6:**
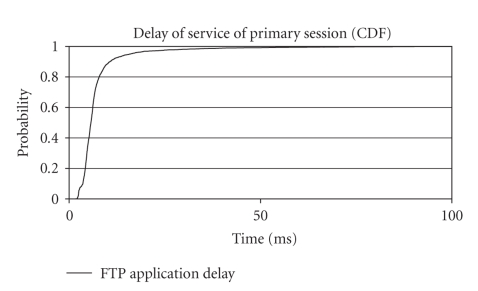
Application delay for third scenario (CDF).

**Figure 7:**
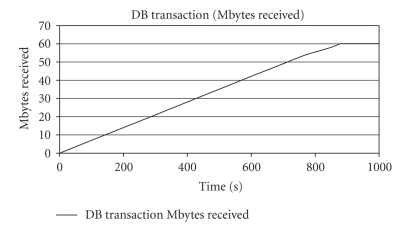
DB transaction (received MBytes) versus time for third scenario.

In this simulation run, the feasibility of mobile access to electronic health records via an enhanced uplink network was examined. The main attributes of this run are summed up in Table 10 and afterwards Figures 6 and 7 depict the results of this simulation. The 60 MB of data from the database were distributed as follows: 100 KB for demographic data, 1 MB for laboratory, clinical data, and medical history, and the rest is medical image and diagnostic video data, according to Table 5.

This scenario was the least demanding among the ones studied. Indicative of this statement is that for the 95% of the samples the delay did not exceed 15 milliseconds, while the equivalent value for the 98% was 30 milliseconds. The services of our primary session were background (FTP and DB transaction) and do not require a great quantity of dedicated resources.

The key feature of this simulation run was the behavior of the DB transaction service. Undoubtedly, 60 MB is a significant capacity of data, taking up a lot of time to be acquired (around 15 minutes, at a speed very close at the maximum allocated per session). In the previous scenario, where we came across the same problem, we assigned a separate session for the DB transaction to be accomplished. At this point, the particular solution would not yield any significant gain due to the massive amount of data. To achieve a decent time (in the range of 4-5 minutes), the equivalent speed would rise above 1.5 Mbps, which is rather inapplicable, at least for the time being in a real enhanced uplink platform. Conclusively, although the system under examination was able to serve this scenario, the time needed was impractical. Considering the low QoS demanded by background services, one may either compromise with this feature, or try a different angle of approach by selecting another system for the massive transmission of background data. WiMAX or WiLAN platforms are ideal candidates in order to cooperate with enhanced uplink and solve this issue.

## 6. CONCLUSIONS

In the present paper, a high-speed 3.5G simulation scheme for serving mobile healthcare applications is studied. The paper demonstrates the feasibility of utilizing a realistic enhanced uplink network for such applications on a number of test cases, that is, emergency health services, teleconsultation collaborative sessions between moving physicians, and mobile access to electronic health records. Transmission of voice, real-time video, biosignals and transfer of files has been taken into account. The performance of the proposed scheme has been evaluated mainly in terms of delay under different load and services requirements. The simulation campaign proved that a careful system design is indispensable in order the system to have the ability to sufficiently cope with the supplied load.

Summarizing the provided results, it was evident that enhanced uplink successfully coped with all the test cases examined. The end-to-end delays in all cases were quite satisfactory, apart from the first test case of the first scenario. This particular simulation run proved that assigning high bit rate to a single session leads to a deterioration of the overall system's performance. To face this issue and to optimally manage the network's resources, the maximum allocated bit rate per session was bounded to 768 kbps and the maximum proposed video bit rate was 512 kbps, unless better quality may be explicitly required. Derived by the second scenario and driven by the fact that EHR transaction services need to be served within a reasonable amount of time, the necessity of a separate session to be occupied by this kind of services was obvious. Finally, although enhanced uplink was able to serve all types of file transfers, massive file transfers induce significant delays for their accomplishment. This remark reinforces the prospect of a collaboration of enhanced uplink with another platform, such as WiMAX, to achieve the improvement of the provided quality of service.

Conclusively, enhanced uplink managed to meet the requirements for remote healthcare applications, serving adequately the generated load. Consequently, it can serve as a new generation technology for mobile health systems providing immediate and ubiquitous health care in a range of different circumstances, as it may handle a variety of telemedicine needs, especially in the fields of emergency health care provision in ambulances, rural hospital centers or any other remote and dispersed located health center, and intensive care patients monitoring. As a future work, the proposed scheme could be evaluated in a more real-life experimental scenario, where an adequate number of users can access the HSUPA network. The above scenario incorporates multicell planning, where the location of the users can be chosen so as to evaluate the effect of mutual interference, handover, and power control problems. Part of future investigation as well could be the integration of the standard H.264/MPEG-4 AVC in the simulation procedure, which achieves significantly higher compression and may partly alleviate some of the described problems. Furthermore, in order the results to be more reasonable for the forthcoming networks, we would like to provide further experimentation facts considering medical data provision over heterogeneous access networks like 802.16.

## Figures and Tables

**Table 1:** Enhanced uplink parameters.

Enhanced uplink category	Max uplink speed
Category 1	0.73 Mbps
Category 2	1.46 Mbps
Category 3	1.46 Mbps
Category 4	2.93 Mbps
Category 5	2.00 Mbps
Category 6	5.76 Mbps
Category 7 (3GPP release)	11.5 Mbps

**Table 2:** Classification of m-health—QoS requirements.

Application type	Required throughput	Small delay	Small jitter
Teleconsultation	High/medium	Yes	Yes
Telediagnosis	High	Yes	No
Telemonitoring	Low/medium	No	No
Teleeducation VoD	High	No	No
Access to EHR	Low/high	No	No

**Table 3:** Typical medical data in emergency services in case of accidents.

Digital device	Signal or image resolution		Data rate required
	Temporal/spatial (no. of samples per second)	Contrast/resolution (bits per sample)	
Location information	1	×16	<10 kbps
Digital blood pressure monitor (sphygmomanometer)	1	×16	<10 kbps
Digital thermometer	5	×16	<10 kbps
Respiration	50	×6	<10 kbps
Digital audio stethoscope (heart sound)	10000	×12	∼120 kbps
Electrocardiogram ECG (3 leads)	1250	×12	∼15 kbps
VoIP communication of paramedics with hospital experts	—	—	∼64 kbps
Compressed and full motion video (telemedicine)	—	—	384 kbps to 1.544 Mb/s (speed)

**Table 4:** Typical medical data teleconsultation collaborative sessions between moving physicians.

Digital device	Signal or image resolution		Data rate required
	Temporal/spatial (no. of samples per second)	Contrast/resolution (bits per sample)	
Demographic data	—	—	100 KB (text size)
Laboratory and clinical data, medical history	—	—	1 MB (text size)
Digital blood pressure monitor (sphygmomanometer)	1	×16	<10 kbps
Digital thermometer	5	×16	<10 kbps
Respiration	50	×6	<10 kbps
Digital audio stethoscope (heart sound)	10000	×12	∼120 kbps
Electrocardiogram ECG	1250	×12	∼15 kbps
Electroencephalogram EEG	350	×12	∼10 kbps
Electromyogram EMG	50000	×12	∼600 kbps
Ultrasound, cardiology, radiology (DICOM)	512 × 512	×8	256 KB (image size)
Magnetic resonance image (DICOM)	512 × 512	×12	384 KB (image size)
Scanned X-ray (DICOM)	1024 × 1250	×12	1.8 MB (image size)
Digital radiography (DICOM)	2048 × 2048	×12	6 MB (image size)
Mammogram (DICOM)	4096 × 4096	×12	24 MB (image size)
Medical video for teleconsulation (e.g., ophthalmoscope, proctoscope, etc.)	—	—	1.544 Mb/s
Voice/video/chat communication of commuting physicians	—	—	384 kbps to 1.544 Mb/s
Common workspace management controls	—	—	∼10 kbps

**Table 5:** Typical medical data in medical information management service.

Digital device	Signal or image resolution		Data rate required
	Temporal/spatial (no. of samples per second)	Contrast/resolution (bits per sample)	
Demographic data	—	—	100 KB (text size)
Laboratory and clinical data, medical history	—	—	1 MB (text size)
Digital audio stethoscope (recorded heart sounds)	10000	×12	∼120 kbps
Ultrasound, cardiology, radiology (DICOM)	512 × 512	×8	256 KB (image size)
Magnetic resonance image (DICOM)	512 × 512	×12	384 KB (image size)
Scanned x-ray (DICOM)	1024 × 1250	×12	1.8 MB (image size)
Digital radiography (DICOM)	2048 × 2048	×12	6 MB (image size)
Mammogram (DICOM)	4096 × 4096	×12	24 MB (image size)
Recording of endoscopic video	—	—	384 kbps Mb/s (speed)

**Table 6:** Fundamental simulator's parameters.

Parameter	Value
Carrier frequency	2.1 GHz
Channel bandwidth	5 MHz
TTI duration	2 msec
Cell radius	800 m
Path loss model	L = 128.1 + 37.6*log_10_R
Slow fading model	Log-normal distribution
Deviation of slow fading	8.0 dB
Thermal noise density	−174 dBm/Hz
Other-to-own-cell interference	0.5
UE TX power	19 to 23 dBm
Rise over thermal (RoT)	6 dB
Traffic generator	VoIP, video, web browsing, FTP
Mobility model	Dense urban vehicular and pedestrian

**Table 7:** Parameters of the first test case of simulation run of the first scenario.

Parameter	Value
Number of sessions	2 (using the same services)
Session's duration	constant (100 seconds)
Services (nominal bit rate kbps)	VoIP (12.2), MPEG (768), FTP (150)

**Table 8:** Parameters of the second test case of the simulation run of the first scenario.

Parameter	Value
Number of sessions	3
Session's duration	constant (100 secs)
Services (nominal bit rate kbps) for first session	VoIP (12.2), MPEG (768), FTP (150)
Services (nominal bit rate kbps) for second and third sessions	VoIP (12.2), FTP (384)

**Table 9:** Parameters of the second scenario.

Parameter	Value
Number of sessions	3
Session's duration	constant (1500 secs)
Services (nominal bit rate kbps) for first and second session	VoIP (12.2), MPEG (512), Controls (10), DB transaction (27 MB)
Services (nominal bit rate kbps) for third session	VoIP (12.2), FTP (256)

**Table 10:** Parameters of the third scenario.

Parameter	Value
Number of sessions	4
Session's duration	constant (1000 secs)
Services (nominal bit rate kbps) for first session	FTP (120), DB transaction (60 MB)
Services (nominal bit rate kbps) for second and third sessions	MPEG (384)
Services (nominal bit rate kbps) for fourth session	VoIP (12.2), MPEG (256)
